# Establishing a Bioink Assessment Protocol: GelMA and Collagen in the Bioprinting of a Potential *In Vitro* Intestinal Model

**DOI:** 10.1021/acsbiomaterials.5c00034

**Published:** 2025-03-25

**Authors:** Mariangela Rea, Luana Di Lisa, Giorgia Pagnotta, Nunzia Gallo, Luca Salvatore, Federica D’Amico, Noelia Campilio, José Manuel Baena, Juan Antonio Marchal, Arrigo F.G. Cicero, Claudio Borghi, Maria Letizia Focarete

**Affiliations:** † Department of Chemistry ‘Giacomo Ciamician’ and INSTM UdR of Bologna, University of Bologna, 40129 Bologna, Italy; ‡ Department of Engineering for Innovation, University of Salento, 73100 Lecce, Italy; § Typeone Biomaterials S.r.l., Via Europa 167, 73021 Calimera, Lecce, Italy; ∥ Department of Pharmacy and Biotechnology, University of Bologna, 40126 Bologna, Italy; ⊥ REGEMAT 3D S.L., 18016 Granada, Spain; ¶ BRECA Health Care S.L., 18016 Granada, Spain; □ Department of Human Anatomy and Embryology, Faculty of Medicine, University of Granada, 18016 Granada, Spain; ■ BioFab i3D Lab, Centre for Biomedical Research (CIBM), University of Granada, 18016 Granada, Spain; ○ Instituto de Investigación Biosanitaria ibs.GRANADA, 18016 Granada, Spain; ● Excellence Research Unit “Modeling Nature” (MNat), University of Granada, 18071 Granada, Spain; △ Medical and Surgery Sciences Department, University of Bologna, 40138 Bologna, Italy; ▲ Cardiovascular Medicine Unit, IRCCS AOU di Bologna, 40138 Bologna, Italy; ▽ Interdepartmental Center for Industrial Research in Health Sciences and Technologies, University of Bologna, Via Tolara di Sopra, 41/E, 40064 Ozzano Emilia, Bologna, Italy; ⬡ Biofabrication group, Department of Pharmacy, School of Health Sciences, Universidad Cardenal Herrera-CEU, CEU Universities, 46115 Alfara de Patriarca, Valencia, Spain

**Keywords:** GelMA, collagen, 3D bioprinting, intestine bioprinted model, rheology

## Abstract

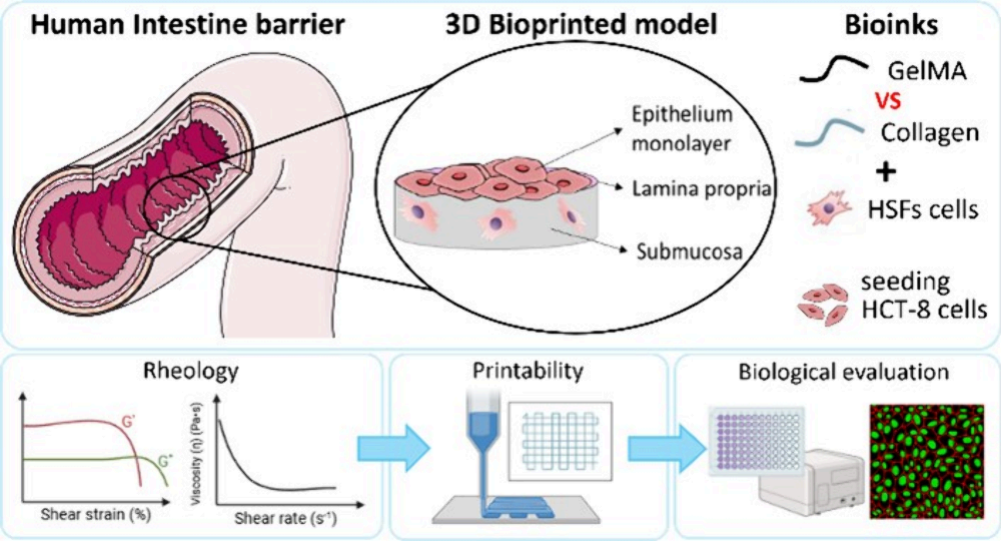

Collagen and gelatin methacryloyl (GelMA) are widely studied biomaterials for extrusion-based bioprinting (EBB) due to their excellent biological properties and ability to mimic the extracellular matrix of native tissues. This study aims to establish a preliminary workflow for approaching EBB by assessing collagen and GelMA printability and biological performance. GelMA was selected for its cost-effectiveness and ease of synthesis, while our collagen formulation was specifically optimized for printability, which is a challenging aspect of bioprinting. A parallel evaluation of their printability and biological performance is provided to develop a preliminary 3D intestinal model replicating the submucosa, lamina propria, and epithelial layer. Rheological analyses demonstrated that both materials exhibit a shear-thinning behavior. Collagen (u-CI) displayed a shear-thinning parameter *p* = 0.1 and a consistency index *C* = 80.62 Pa·s, while GelMA (u-GI) exhibited a more pronounced shear-thinning effect and enhanced shape retention (*p* = 0.06, *C* = 286.6 Pa·s). Post-extrusion recovery was higher for collagen (85%), compared to GelMA (45%), indicating its greater mechanical resilience. Photo-crosslinking improved hydrogel stability, with an increase in storage modulus *G*′ for both materials. Printing tests confirmed the suitability of both hydrogels for bioprinting, with GelMA demonstrating higher print fidelity than collagen. Dimensional stability assessments under incubating conditions revealed that collagen constructs maintained their shape for 14 days before degradation, whereas GelMA constructs exhibited a gradual decrease in diameter over 21 days. Cell culture studies showed that human skin fibroblasts (HSFs) and human colon adenocarcinoma cells (HCT-8) could be successfully cocultured in an optimized RPMI 1640-based medium. AlamarBlue assays and Live/Dead staining confirmed high cell viability and proliferation within both hydrogel matrices. Notably, HSFs in GelMA exhibited more elongated morphologies, likely due to the material’s lower stiffness (380 Pa) compared to collagen (585 Pa). HCT-8 cells adhered more rapidly to GelMA constructs, forming colonies within 7 days, whereas on collagen, colony formation was delayed to 14 days. Finally, a layered intestinal model was fabricated, and immunostaining confirmed the expression of tight junction (ZO-1) and adhesion (E-cadherin) proteins, validating the epithelial monolayer integrity. These findings highlight the potential of collagen and GelMA in 3D bioprinting applications for gut tissue engineering and pave the way for future developments of *in vitro* intestinal models.

## Introduction

Bioprinting is gaining interest in tissue engineering,^1^ toxicity testing, drug discovery,^2^ and organ-on-a-chip platforms.^3^ It enables the codeposition of cells and biomaterials (i.e., what is called a bioink) in complex microstructures with fine spatial control. The three-dimensional (3D) bioprinted constructs accurately reflect the histological and physiological complexity of real tissues by simulating cell-microenvironment interactions.^4−6^ Extrusion-based bioprinting (EBB) enables the rapid fabrication of 3D scaffolds by continuously depositing layers of bioink filaments.^7^ Due to its broad viscosity range processability (from 30 to 6 × 10^7^ mPa·s), multi-material printing capability, and suitability for high cell densities,^8^ EBB remains one of the most used bioprinting techniques.^9^ However, it provides lower resolution than other methods, limiting its ability to accurately replicate fine *in vivo* microstructures. Hydrogels used in bioink formulation must be biocompatible, biodegradable, and mechanically suitable.^10^ Among these, rheological properties critically influence printability and shape fidelity.^11^ The ideal bioink should exhibit (i) a gel-like state before dispensing (ii) shear-thinning behavior during extrusion and (iii) structural retention postdeposition. Therefore, evaluating hydrogel viscoelasticity is essential in bioink development alongside cell viability and proliferation assessments.

Commonly used biomaterials for bioprinting include natural polymers such as alginate, gelatin, collagen, hyaluronic acid, chitosan, and decellularized extracellular matrices (d-ECMs), as well as synthetic polymers, including poly(ethylene glycol) (PEG), Pluronic, poly(vinyl alcohol) (PVA).^12^ Among these, collagen is particularly relevant due to its abundance in the human body, high biocompatibility, and low immunogenicity, which have supported its widespread biomedical use.^13^ Collagen is the major insoluble fibrous protein in the ECM and connective tissues, encoded by a large family of genes. Twenty-eight different types of collagen have been identified, and classified based on their gene sequences, structural organization, and tissue distribution.^14,15^ Its application in 3D bioprinting is challenging due to low mechanical strength, limited mass transport, and structural instability. To overcome these limitations, fibrillar collagens have been used instead of the soluble collagen forms. Fibrillar collagens, such as type I, exhibit a characteristic triple-helical structure that self-assembles into fibrils under physiological conditions. Typically, fibrillar collagens require weakly acidic conditions for resuspension and printing, which are not cell compatible. A recently developed equine tendon-derived fibrillar collagen bioink formulation (see Materials and Methods section) overcomes these challenges, enabling 3D structures with tunable mechanical properties in physiologically relevant conditions. Methacrylated gelatin (GelMA) is another widely used biomaterial in bioinks. Derived from collagen, gelatin undergoes thermoreversible gelation, but methacrylation allows for chemical crosslinking, providing enhanced stability. GelMA is easily synthesized and crosslinked with high precision, allowing the tuning of mechanical property based on application requirements while maintaining biocompatibility.^16,17^

The intestinal barrier plays a fundamental role in digestion, acting as a selective barrier, and it is responsible for the absorption of water and electrolytes and the elimination of body wastes.^18^*In vitro* models of the intestinal barrier allow for an in-depth and accurate study of physiopathological processes, as well as drug testing. Efforts have been made to develop 3D bioprinted intestinal models with appropriate hierarchical architecture and cell composition.^19−21^ Biomaterials such as collagen,^20^ decellularized matrices,^22^ GelMA,^23^ alginate,^24^ and PEG diacrylate^25,26^ have been explored. However, recapitulating the complex structure of intestinal villi^27,28^ and cellular composition remains challenging, especially through EBB, due to its limited resolution compared to other bioprinting technologies.

In this work, a simplified bioprinted intestinal model was developed to replicate the three fundamental layers of the intestinal barrier: the submucosa, the lamina propria, and epithelial layer.^29^ Given the resolution limitations of the EBB, the model does not aim to replicate specific villi microstructures but provides a structured platform for intestinal tissue modeling. Specifically, the submucosa layer was printed by including patient-derived human skin fibroblasts (HSFs) in the bioinks, the lamina propria was printed without cells, and the epithelial layer was recreated by culturing the human colon epithelial cell line (HCT-8) on the 3D bioprinted construct. These cell types were selected as biological models to preliminarily assess the suitability of the materials and EBB technology for intestinal barrier modeling. Collagen and GelMA hydrogels were chosen for bioink formulation due to their widespread use in the scientific community, ensuring broad applicability. Their morphological, rheological, and biological properties were investigated to highlight the strength of each material in the bioprinting process. This work seeks to introduce a detailed protocol for developing a bioink focusing on printability, shape fidelity, and cell viability, while proposing a preliminary *in vitro* intestinal model, designed for easy adoption and application to various hydrogels in the EBB process. Beyond proof-of-concept, the proposed bioprinted model bridges the gap between oversimplified 2D systems and clinical applications, offering a versatile platform for future studies of barrier function, permeability, metabolism, transport, and toxicity.

## Materials and Methods

### 2.1 Collagen Hydrogel Preparation

The collagen ink (CI), made of fibrillar type I collagen (native form) from an equine tendon, was produced according to a Typeone Biomaterials Srl proprietary process and provided as a 25 mg/mL ready-to-use sterile suspension in neutral buffered solution (pH 7.4). No crosslinking agents were present nor added. Photo-crosslinking of CI is achieved through UV light irradiation (365 nm) with 12 mW/cm^2^ intensity for 2 min at a 3 cm distance between the sample and the irradiating lamp.

### 2.2 Gelatin-Methacrylate Synthesis and Hydrogel Preparation

Type A gelatin from porcine skin (gel strength 300), methacrylic anhydride (MAA) (94%), sodium carbonate, sodium bicarbonate, and dialysis cellulose membranes (12–14 kDa cutoff avg. flat width 25 mm) were purchased from Sigma-Aldrich. GelMA ink was produced according to a previously optimized protocol.^30^ Briefly, gelatin (10% w/v) was dissolved in a 0.25 M carbonate-bicarbonate buffer (pH 9). Subsequently, an excess of MAA (0.1 mL/g gelatin) was dropped into the gelatin solution under vigorous stirring (500 rpm) at 55 °C. Once the reaction started, the formation of methacrylic acid subproduct resulted in a pH decrease. For this reason, to promote the methacrylation process, 5 M NaOH was added to the reaction mixture to adjust the pH to 9. After 1 h, the reaction was quenched by adding HCl 37%v/v dropwise until reaching pH 7.4. To remove the methacrylic acid subproduct produced during the reaction and any traces of unreacted MAA, the mixture was dialyzed for 5 days at 37 °C under gentle stirring against ultrapure water (Milli-Q H_2_O). The dialyzed GelMA solution was freeze-dried and stored at −4 °C and protected from the light until further use. The GelMA hydrogel ink (GI) was prepared by dissolving the lyophilized GelMA in phosphate buffer saline (PBS) (1×) at 37 °C at a final concentration of 5% w/v. The hydrogel was prepared inside a 5 mL sterile disposable syringe equipped with a female/female luer lock adapter, enabling the attachment to a second syringe. This double syringe system was then exploited to mix and homogenize the hydrogel. Once GelMA was dissolved, Irgacure 2959 photoinitiator (I2959) (Sigma-Aldrich) at a 0.1% w/v concentration was added to the solution. The GI is photo-crosslinked with UV light irradiation (365 nm) with 0.96 mW/cm^2^ intensity for 3 min at a 3 cm distance between the sample and the irradiating lamp for rheological analysis.

### 2.3 Rheological Characterization

The rheological properties of GI and CI were assessed by means of an MCR 102 parallel-plate rheometer (Anton Paar, Graz, Austria) in a plate–plate geometry with a diameter of 25 mm (PP-25 plate) and a gap of 0.3 mm. All measurements were carried out in triplicate at 20 °C to simulate the static condition during preprinting and postprinting process. The following oscillatory tests were carried out: amplitude sweep, gelation, and an isothermal test. The rotational analysis included flow curves and three interval thixotropic test (3ITT) and were performed in controlled shear rate mode. The input data were set up through the Rheoplus program. About 500 μL of hydrogel was deposited onto the plate of the rheometer by using a syringe. Subsequently, the upper plate was lowered until it came into contact with the surface of the sample. The excess material out of the plates was removed with a spatula, and the trap was filled with distilled water to avoid evaporation.

#### Amplitude Sweep

Amplitude sweep analyses were performed on both GI and CI before and after crosslinking in a strain range (γ) from 0.01% to 1000% by keeping the frequency constant at 1 rad/s. This test allows the evaluation of the storage modulus (*G*′) and the loss modulus (*G*″) as a function of the applied strain (%), the determination of the linear viscoelastic range (LVE), and the crossover point (*G*′ = *G*″) which provides a measure of the stress needed to induce the flow of macromolecular chains within the hydrogel. All of the successive tests were carried out within the LVE.

#### Gelation

This test was exploited to evaluate how the sample’s rheological properties varied as a function of temperature. The angular frequency and strain amplitude were kept constant at 1 rad/s and 0.1%, respectively, to mimic static conditions. The starting temperature was set to 40 °C while the final one was set to 4 °C, and a linear ramp of 5 °C/min was applied. This test allowed us to identify the temperature interval in which the gel-like behavior of the samples is present, which is important to define printing parameters such as the printhead and substrate temperatures.

#### Isothermal Test

An isothermal test is useful to evaluate how the *G*′ and *G*″ change over time, by keeping constant the strain amplitude (0.1%), angular frequency (1 rad/s), and temperature (37 °C). This measure was performed after the crosslinking process, to evaluate its effectiveness. The time was set to 10 min, and the temperature of 37 °C was selected to simulate the incubating conditions to which the printed constructs will be subjected after the printing.

#### Flow Curves

Flow curve tests were performed in the range of shear rates (*γ̇*) from 0.1 to 1000 s^–1^, with a “ramplog + decade” profile and a slope of 6. The flow curves are a plot of the viscosity (η) or the shear stress (τ) as a function of the applied shear rate. The obtained plots were fitted according to the Ostwald-Waele mathematical model (eq 1) to obtain the shear thinning index (*p*) and the consistency index (*C*).
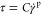
1

#### Three-Interval Thixotropic (3ITT) Test

3ITT was exploited to simulate the extrusion-based 3D-bioprinting process and to assess the hydrogel’s mechanical properties after the application of high shear rates. Specifically, the measure was performed with controlled shear rate (CSR) mode, and the resulting graph reports the viscosity as a function of time. The rest condition of the hydrogel inside the syringe, prior to the printing, was simulated by applying a low shear rate (0.1 s^–1^) at 20 °C. Then, a high shear rate was applied to simulate extrusion through the nozzle of the printing syringe. This value was calculated through eq 2.
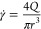
2where *Q* is the volumetric flow rate and *r* is the value of the nozzle radius. *Q* for a cylindrical nozzle was determined using eq 3:
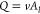
3where *A*_l_ is the lateral area of the section and *v* represents the flow velocity.

### 2.4 Cell Culture

#### Materials

Dulbecco’s modified Eagle medium (DMEM), Gibco Roswell Park Memorial Institute 1640 (RPMI 1640), GlutaMAX, fetal bovine serum (FBS), penicillin/streptomycin solution, sodium pyruvate, horse serum (HS), and trypsin were provided by Thermo Fisher Scientific Co LLC (Waltham, Massachusetts, USA). If not otherwise stated, all other chemicals used were of analytical grade and purchased from Merk.

#### HSF Cell Culture

Human skin fibroblasts (HSFs) were isolated from skin tissue obtained during abdominoplasty surgery (ethics committee reference 0467-N-20) after obtaining written informed consent, following a well-established protocol.^31^ Cells were cultured with DMEM supplemented with GlutaMAX, 10% FBS, and an antibiotic/antimycotic solution consisting of 100 U/ml penicillin and 100 μg/mL streptomycin in culture flasks under controlled conditions (5% CO_2_ and 37 °C). The culture media was changed every 2 days. HSFs at passages 4–6 were characterized by flow cytometry for mesenchymal and hematopoietic surface markers (CD73, CD90, CD105, CD34, CD45, and HLA-DR), following the established criteria of the International Society for Cellular Therapy.^32^

#### HCT-8 Cell culture

Human colonic epithelial (HCT-8) cell line was provided by the Center of “Instrumentación Científica” from the University of Granada. HCT-8 cells were cultured in RPMI 1640 supplemented with 2 mM Glutamine, 1 mM sodium pyruvate, 5% HS, 5% FBS, and penicillin-streptomycin (at concentrations of 100 U/ml and 100 μg/mL, respectively) in cell culture flasks and maintained at 5% CO_2_ and 37 °C. The culture media was changed every 2 days.

#### HSF and HCT-8 Cell Cocultures

Human skin fibroblasts (HSFs) and Human colonic epithelial (HCT-8) cell lines were cocoltured in RPMI 1640 supplemented with 5% of FBS and 5% of HS in cell culture flasks and maintained at 5% CO_2_ and 37 °C.

### 2.5 3D Bioprinting

**Scheme 1:**
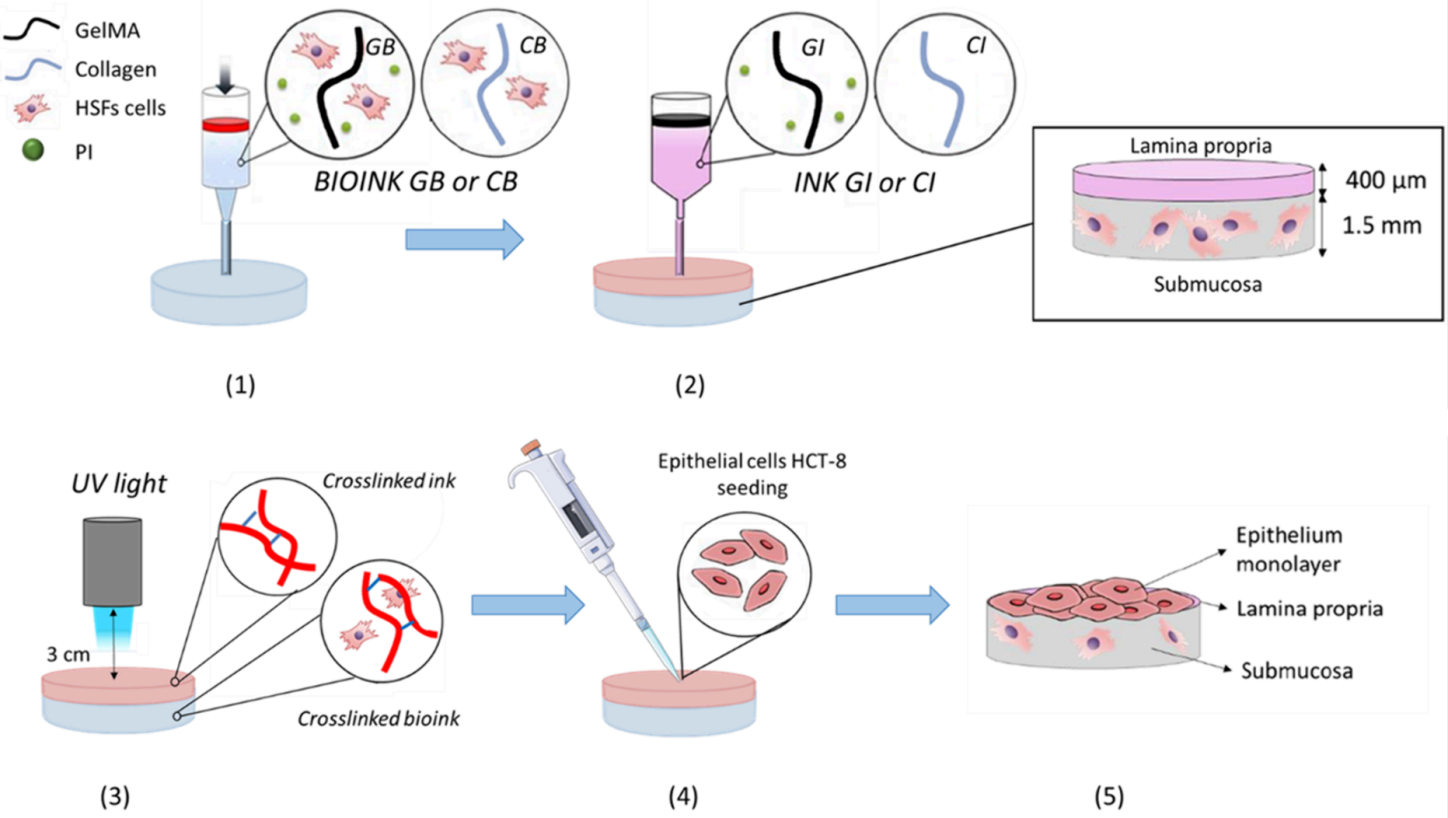
Bioprinting Process of the Intestinal Model: (1) Bioprinting of GelMA Bioink (GB) or Collagen Bioink (CB) Containing HSFs Cells to Reproduce the Submucosa Layer; (2) Printing of GelMA Ink (GI) or Collagen Ink (CI) to Reproduce the Lamina Propria Layer; (3) UV Crosslinking of GelMA or Collagen Chains within the Bioink and the ink; (4) HCT-8 Cells Seeding on the Top Layer; (5) Formation of the Epithelium Monolayer

The conceived model seeks to reproduce the layered structure of the intestinal interface. Collagen bioink (CB) and GelMA bioink (GB) were prepared in a 1:10 (v/v) ratio by mixing 100 μL of culture medium containing 10^6^ cells with 1 mL of the respective ink. Bioinks were transferred to the bioprinting syringe barrels equipped with highly adjusted pistons and 0.41 mm ID conical tips and placed in the corresponding printheads. All the constructs were printed by following the predesigned geometry inside a 24-multiwell. Following extensive optimization, a nozzle diameter of 0.41 mm, a flow speed of 3 mm/s, a perimeter speed of 10 mm/s, and an infill speed of 10 mm/s were selected for printing both bioinks. The printing temperature was maintained at 20 °C to prevent nozzle clogging caused by the thermo–responsive nature of GelMA and Collagen. Two syringe printheads were employed for the intestinal model fabrication, and the bioprinting steps are illustrated in Scheme 1. One syringe was filled with either CB or GB containing HSF cells to obtain a submucosal layer with circular shapes (0.5 mm in thickness and 10 mm in width). The second syringe was filled with either CI or GI to print the lamina propria layer (0.4 mm in thickness and 10 mm in width) above the previous layer. Specifically, the CI was printed above the CB layer, and the GI was printed above the GB layer. After the printing process, collagen in the CB was photo-crosslinked with UV light irradiation (365 nm) with 12 mW/cm^2^ intensity for 2 min at a 3 cm distance between the sample and the irradiating lamp. Similarly, the photo-crosslinking process of GelMA in GB was carried out through UV light irradiation (365 nm) with 0.96 mW/cm^2^ intensity for 3 min at a 3 cm distance between the sample and the irradiating lamp. Different light intensities were applied to the materials due to the differing photo-crosslinking mechanisms: collagen photo-crosslinks without a photoinitiator, driven by a transition in its triple helix structure, whereas GelMA requires a photoinitiator to form lateral covalent bonds, thus needing less energy. The bioprinter Bio V1 (REGEMAT 3D S.L., Granada, Spain) equipped with an extrusion-based piston-driven system was used under a laminar flow hood. The 3D constructs were designed by using a CAD design model integrated with the custom-made bioprinter’s software. The CAD models were exported as Standard Triangle Language (STL) files to generate the G-Coded files. Finally, to create the outermost epithelial monolayer, HTC-8 cells were manually seeded onto the surface of the printed scaffolds.

### 2.6 Cell Response

#### Materials

Hoechst 33342, phosphate-buffered saline (PBS), Propidium Iodide (PI), Calcein-AM, paraformaldehyde (PFA), and Triton X-100 were purchased from Merk KGaA (Darmstadt, Germany). AlamarBlue reagent was provided by Thermo Fisher Scientific Co LLC (Waltham, Massachusetts, USA). Primary antibodies rat anti- ZO-1 conjugated with AlexaFluor 488 and mouse anti-E-cadherin were purchased from Santa Cruz Biotechnology (Dallas, Texas, USA). Goat antimouse secondary antibody (AlexaFluor 594) was acquired from Jackson ImmunoResearch Inc. (Ely, United Kingdom).

#### Cell Proliferation

The alamarBlue assay was carried out to estimate the cell proliferation after the 3D bioprinting process up to 21 days, following manufacturer’s instructions. Given that alamarBlue measures the whole cell metabolic activity in the coculture with no discrimination between cell types, 3D bioprinted constructs containing either single cultures of HSFs or HCT-8 were used for this assay. Briefly, three biological replicates per group were incubated with 10% v/v alamarBlue solution in cell culture medium for 4 h at 37 °C in a cell culture incubator, protected from direct light and following manufacturer’s instructions. Then, fluorescence intensity was measured in three technical replicates per sample at an excitation wavelength of 530 nm and emission of 590 nm (Microplate Reader MB-580/30, Heales). The intensity of the fluorescent signal was expressed in relative fluorescence units (RFU). For each measurement, the corresponding tissue-engineered construct without cells (i.e., CI and GI) was measured under identical conditions to determine the baseline fluorescent signal. The blank value was then subtracted from the cellularized construct values to obtain the fluorescent signal originating from the cellular activity. RFU values were normalized as a fold increase to day 1.

#### Cell Viability

Live/dead assay was performed to evaluate cell viability through confocal imaging for up to 21 days. At prefixed time points (7, 14, 21 days), 3D cell cultures were washed three times with 1 mL of PBS (1X) before staining with 0.5 mL of live/dead solution (0.33 μM Calcein-AM and 2 μM PI), containing Hoechst 33342 at a concentration of 4 μg/mL, at 37 °C with 5% of CO_2_ for 30 min. Then, the staining solution was removed, and the 3D cell cultures were rinsed three times with 1 mL of PBS (1X) and observed with LSM 710 confocal microscope (Zeiss, Jena, Germany) employing three laser lines (405, 488, and 543 nm) and three detection PMTs (408–498 nm, 493–542 nm, and 554–738 nm) for the blue, green, and red false color channels, employed to measure the fluorescence intensity of 33342 Hoechst, Calcein-AM and PI, respectively. Cell-free scaffolds were used as controls. Fresh samples were used at each time point to prevent potential cytotoxic cumulative effects of the reagents employed.

#### Immunofluorescence

Immunofluorescence analyses were carried out on collagen and GelMA-based 3D cell cultures displaying HCT-8 epithelial monolayers for up to 21 days to confirm the integrity of the epithelial layer. The presence of tight and adherent junctions was confirmed using specific antibodies to detect Zonula occludens-1 (ZO-1), one of the scaffolding proteins that link tight junction transmembrane proteins such as claudins, junctional adhesion molecules, and occluding to the actin cytoskeleton of all tight junctions, and E-cadherin (E-cad), the core component of epithelial adherent junctions. Immunostaining was performed as described previously.^33^ Briefly, samples were rinsed with prewarmed PBS, fixed in 4% PFA dissolved in PBS (pH 7.4) for 30 min, washed with PBS, and blocked with blocking solution for 45 min. Subsequently, samples were incubated with primary antibodies against ZO-1 (1:50 dilution) and E-cad (1:100 dilution) in an incubation buffer overnight at 4 °C. Then, samples were incubated with AlexaFluor 594 secondary antibody (1:150 dilution) for 1 h at room temperature to enable its reaction with E-cad primary antibody. Finally, to visualize cell nuclei, a staining with the Hoechst 33342 solution (1:1500 dilution) was performed.

## 3 Results and Discussion

Owing to their favorable biological and mechanical properties, collagen and GelMA are deeply studied as biomaterials in EBB. In this work, a parallel study of their printability and biological properties was conducted to develop a preliminary intestinal barrier model that can faithfully reproduce the submucosa, the lamina propria, and the epithelial layer structures (Scheme 1), providing an improved point of view focused on accurately replicating the *in vivo* physiognomy.

### 3.1 Rheological Characterization

**Figure 1:**
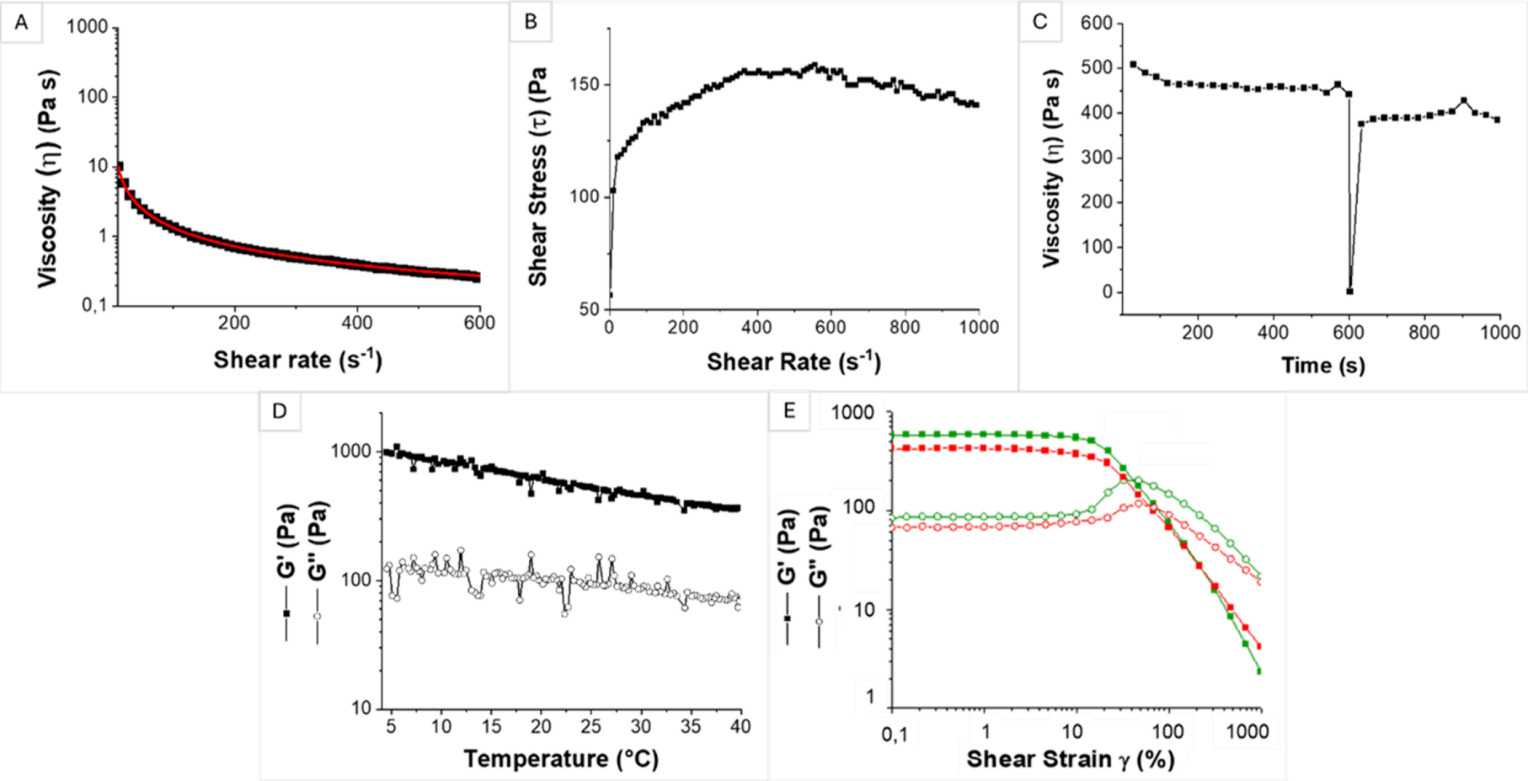
Rheological characterization of collagen hydrogel formulation. (A) Viscosity curve of u-CI fitted with the Ostwald-De Waele model (red line); (B) flow curve of u-CI; (C) three-interval thixotropic test (3ITT) of u-CI; (D) gelation measure of c-CI; (E) amplitude sweep test of u-CI (red curves) and c-CI (green curves).

First, a comprehensive rheological screening was developed and conducted to evaluate the mechanical properties of the materials throughout the printing process. To evaluate collagen’s shear-thinning behavior, which is linked to its printability, a rotational measurement was performed, and the results are presented in Figure 1(A,B), which shows the viscosity (η) and shear stress (τ) as a function of shear. The typical shear-thinning behavior of a printable material was observed since the viscosity decreased by increasing the applied shear rate, from 560 Pa·s at 0.1 s^–1^ to 0.14 Pa·s at 1000 s^–1^. The Ostwald-De Waele regression (eq 1), which quantitatively identifies the shear-thinning behavior of gels, was applied for the medium shear rate range to the obtained viscosity curve (red curve in Figure 1(A)). The shear-thinning parameter (p) and the consistency index (*C*) were derived, resulting in values of 0.11 and 80.62 Pa·s, respectively. They proved the shear-thinning characteristic of un-cross-linked collagen ink (u-CI); indeed, a *p*-value lower than 1 is typical of shear-thinning materials and the *p*-value decreases as this characteristic becomes more pronounced. Regarding the *C* parameter, the higher the *C*, the slower the flow and deformation of the material, preventing the printed structure from collapsing. Therefore, this parameter gives insights into construct’s shape preservation. The obtained *C* value for u-CI is an indication of good shape preservation and durability of the obtained construct. The shear stress vs shear rate graph reported in Figure 1(B) provides an indication of the value of the shear stress corresponding to the specific shear rate (or printing rate of the bioprinting process) that will be experienced by the cells once embedded in the hydrogel. According to eq 2, if the bioprinting process is carried out with a flow rate of 3 mm/s and a nozzle with a diameter of 0.41 mm and a height of 1 mm, then the resulting shear rate is equal to 300 s^–1^. The shear stress corresponding to this shear rate is 150 Pa, which is considered safe for the cells when embedded in the hydrogel.^34,35^

The simulation of the entire printing process was conducted using the 3ITT test, which enables the prediction of how the hydrogel behaves before, during, and after extrusion. A low shear rate (0.01 s^–1^) was selected to simulate the steady static condition both inside the syringe (before extrusion), and after the extrusion process (filament deposited onto the bioprinter bed), while a high shear rate (300 s^–1^) was used to ideally simulate the extrusion process. This value was calculated using eq 2. As reported in Figure 1(C), collagen was characterized by a viscosity of 442 and 375 Pa·s before and after printing, respectively, while during the extrusion, it displayed almost zero viscosity. This behavior demonstrated that collagen hydrogel formulation could be easily extruded at 20 °C, and it almost recovered its initial mechanical properties (85% of recovery after extrusion), being suitable for EBB bioprinting.

A gelation measurement was performed to assess how the rheological properties of u-CI vary with temperature. Specifically, u-CI was exposed to a temperature ramp from 4 to 40 °C, and a slight decrease of the rheological moduli was observed with increasing temperature (Figure 1(D)). The material exhibited gel-like behavior at 20 °C with a *G*′ value of 605 Pa, and *G*″ value of 93 Pa, so 20 °C was selected as optimal printing temperature, although we believe that this formulation can be printed in a wide range of temperatures since the material was able to maintain its gel-like property also at higher temperatures. The viscoelastic behavior of the collagen suspension was evaluated through an amplitude sweep test. As shown in Figure 1(E), u-CI showed a gel-like network since *G*′ was higher (425 Pa) than *G*″ (70 Pa). Furthermore, the LVE range was maintained up to 6% of strain, and the crossover point occurred at 50%, after which the material started to behave as a liquid. The cross-linking process was carried out on collagen to enhance its stability, by exploiting its inherent ability to self-respond to UV light^30^ and further rheological analyses were conducted on cross-linked collagen (c-CI). Photo cross-linking was accomplished using UV light. As shown in Figure 1(D), a slight rise in the *G*′ (585 Pa) and *G*″ (85 Pa) moduli was observed after cross-linking, along with a more pronounced peak of the *G*″ curve, which is indicative of a cross-linked structure. The crossover point was maintained at 50% strain and the LVE ranged from 0.1% to 7%. These findings validate that the application of UV light leads to further cross-linking of collagen chains.

**Figure 2:**
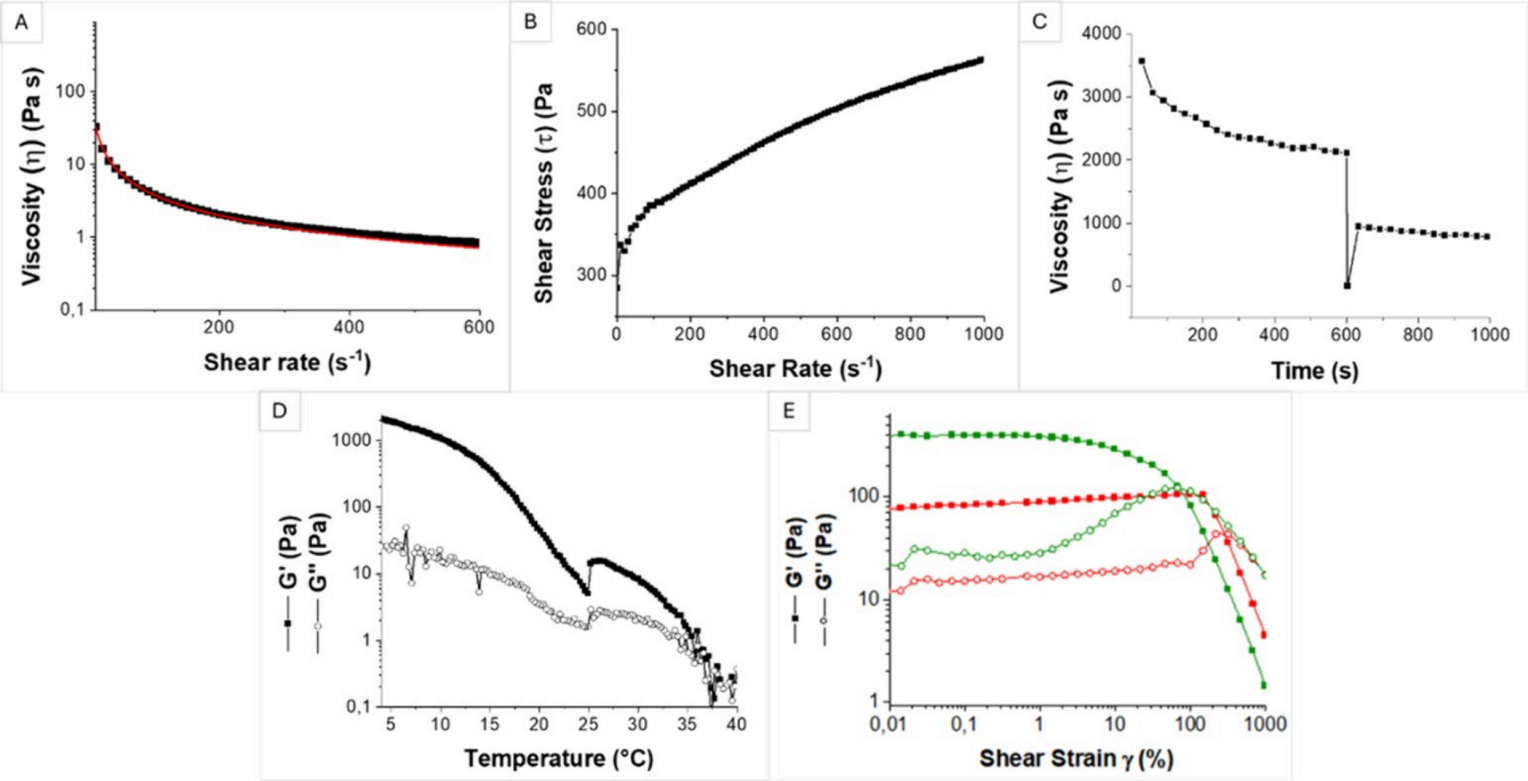
Rheological characterization of GelMA hydrogel formulation. (A) Viscosity curve of u-GI fitted with the Ostwald-De Waele model (red line); (B) flow curve of u-GI; (C) Three-interval thixotropic test (3ITT) of u-GI; (D) gelation measure of c-GI; (E) amplitude sweep test of u-GI (red curves) and c-GI (green curves).

The rheological characterization of the GI formulation was performed to assess printability, mechanical properties, and dimensional stability over time, following the same methodology employed for the CI formulation. The results of viscosity measurements (Figure 2(A)) as a function of shear rate indicated the typical shear-thinning behavior of printable hydrogels, as viscosity decreased from 825 Pa·s at 0.1 s^–1^ to 0.5 Pa·s at 1000 s^–1^. The Ostwald-De Waele regression yielded a p value of 0.06 for u-GI, further confirming its shear-thinning characteristic. Additionally, the C value was determined to be 286.6 Pa·s, a value higher than that found for u-CI. This suggests a greater tendency for shape preservation in GI constructs compared to CI. Moreover, Figure 2(B) provides information on the shear stress that cells will experience upon embedding in the hydrogel as a result of the specific shear rate applied during the bioprinting process. Based on the same calculations of those reported above for u-CI, a shear stress value of 430 Pa was determined for u-GI, which is higher compared to u-CI. Therefore, u-CI bioink is safer for cells’ extrusion compared to u-GI one, although it is important to consider that different cell types can be differently influenced by the application of a certain shear stress during extrusion. When dealing with particularly sensitive cells, it is recommended to use a bioink that is characterized by shear stress values in the same range of those obtained for u-CI formulation.

The results of the 3ITT rheological test, shown in Figure 2(C), revealed that prior to bioprinting, the viscosity exhibited time-dependent behavior at a constant shear rate (0.1 s^–1^), decreasing from 3570 to 2110 Pa·s. After extrusion, this trend persisted, albeit less prominently, with initial and final viscosities of 945 Pa·s and 783 Pa·s, respectively, resulting in a 45% recovery. This suggested that u-GI may not fully regain its mechanical properties post-extrusion due to partial disruption of physical entanglements established at 20 °C.

The behavior of u-GI with temperature, reported in Figure 2(D), demonstrated a decrease in the G′ value from 2090 Pa at 4 °C to 40 Pa at 40 °C, with a crossover occurring between the moduli around 37 °C where the material started acting as a liquid. From this measure, 20 °C was selected as the optimal printing temperature, and the need to cross-link the hydrogel after the printing was highlighted since the final application involves incubation at 37 °C, and without cross-linking, the material would lose its structural integrity. As illustrated in Figure 2(E), u-GI exhibited gel-like behavior with a *G*′ value of 100 Pa and a *G*″ value of 20 Pa, and a crossover point occurring at 286% strain. Following the photo cross-linking process, c-GI displayed an increase in both *G*′ (380 Pa) and G*″* (27 Pa), accompanied by a more pronounced peak in *G*″. This suggested a highly interconnected network due to the presence of (i) physical cross-links at 20 °C and (ii) covalent cross-links formed between acrylate moieties after UV treatment. Furthermore, the shift of the crossover point to a lower strain value (320%) for c-GI indicates a slightly higher rigidity of the material after photo cross-linking.

### 3.2 Printability Study

**Figure 3:**
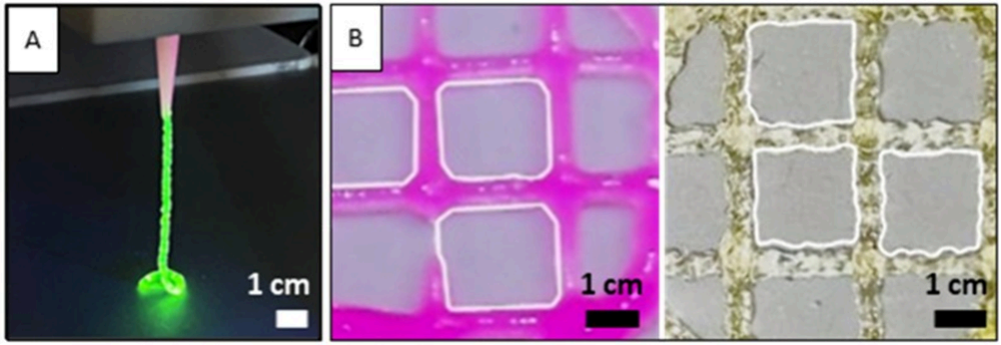
(A) Extruded filament of u-GI stained with fluorescein; (B) printed c-CI (pink) and c-GI (light yellow) grids for the printability and uniformity ratio calculation.

Printing tests were conducted to assess the uniformity ratio and printability (Pr) of the material. The initial qualitative observation regarding hydrogel printability was the preservation of the filament structure after extrusion (Figure 3(A)). Subsequently, quantitative parameters were obtained by printing a round-grid of both hydrogels with a 2 cm diameter, as illustrated in Figure 3(B). The uniformity ratio for c-CI and c-GI was calculated by comparing the expected filament length with its actual length, accounting for its roughness. The resulting uniformity ratio for c-CI (pink grid) was 0.96 ± 0.02, indicating a satisfactory level of uniformity of the printed filament, while the uniformity ratio for c-GI (yellowish grid) was 0.94 ± 0.05, highlighting the higher roughness of c-GI filament compared to collagen. This aspect is valuable in the development of an intestinal barrier model, particularly for replicating the villi-like structure through EBB technology. Indeed, the inherent roughness of the resulting filament can effectively mimic the villi layer, overcoming the resolution limitations of the traditional EBB method. The Pr values, calculated according to eq 4, were found to be 0.91 ± 0.02 for c-CI and 1.01 ± 0.05 for c-GI. In general, a Pr value of 1 indicates a perfect square, reflecting high shape fidelity of the printed construct and maintenance of the extruded filament shape.^36^ In this case, not a perfect square was obtained for c-CI, resulting in a discrete shape fidelity, while for c-GI a Pr value closer to 1 was calculated, indicating higher shape fidelity compared to the collagen grid. All dimensional measurements were conducted in triplicate by using IC measure software.
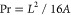
4Lastly, another qualitative printability test was performed by printing a flower pattern using both hydrogels and comparing it with the CAD model (Figure S1(A,B)). The results demonstrated high fidelity of the printed pattern to the CAD model in terms of dimensions and shape, with the c-GI (Figure S1(B)) filament showing higher roughness compared to c-CI (Figure S1(A)). This test was conducted to demonstrate the feasibility of printing more complex shapes with the formulated inks.

The 3D printing parameters for both materials were optimized *ad hoc* to preserve the imprinted shape as much as possible. As a result, the printed materials exhibited distinct mechanical properties. Based on this observation, this study conducted an assessment of collagen and GelMA bioinks for developing an *in vitro* intestinal model.

### 3.3 Dimensional and Mechanical Stability Study under Incubating Conditions

**Figure 4:**
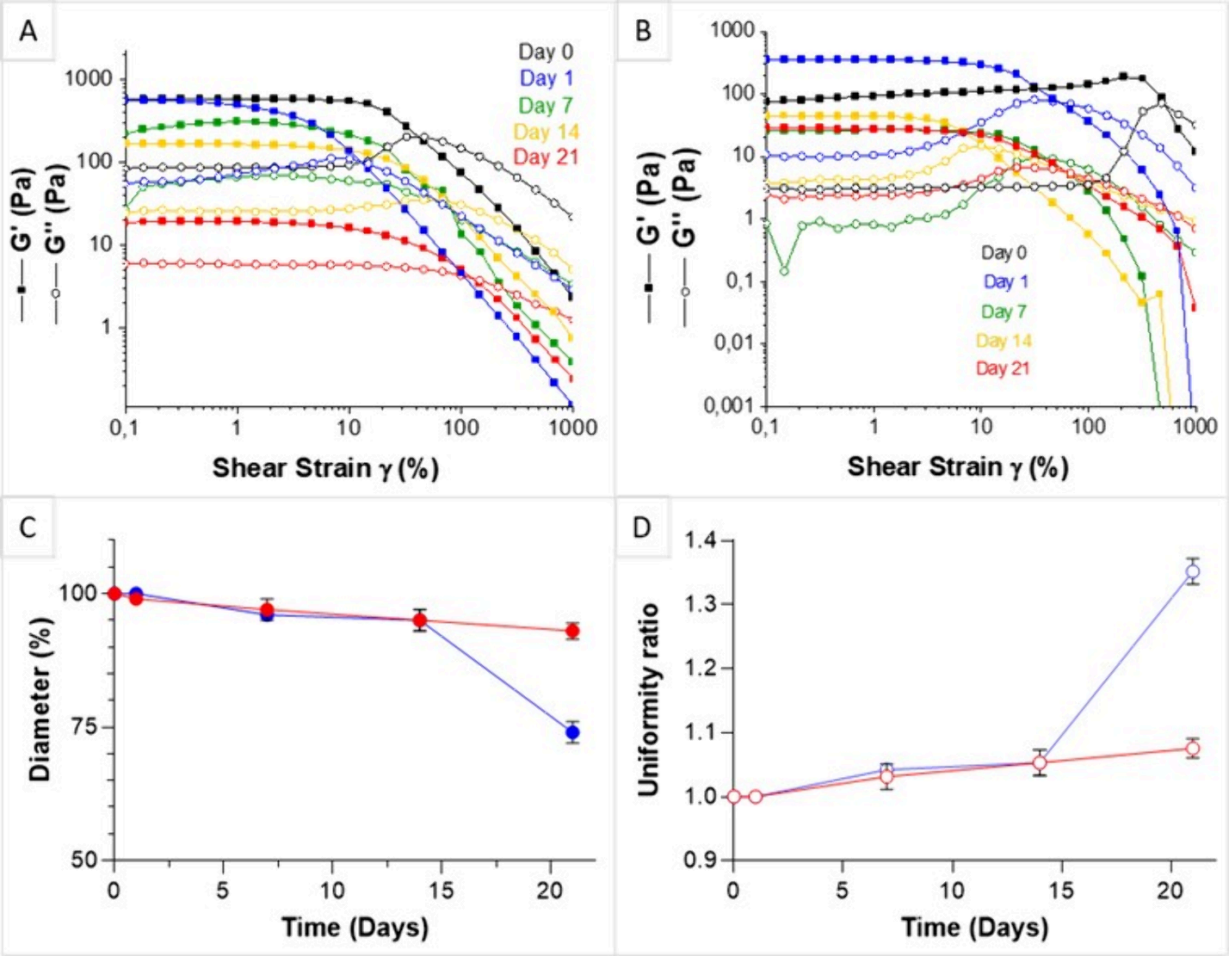
Amplitude sweep analysis of (A) c-CI constructs and (B) c-GI constructs after 0, 1, 14, and 21 days under static incubating conditions. (C) c-CI (blue) and c-GI (red) constructs’ diameter (%) over time under static incubating conditions. (D) Uniformity ratio of c-CI (blue) and c-GI (red) constructs vs time under static incubating conditions.

The dimensional and mechanical stability of printed c-CI and c-GI constructs was assessed under static conditions in PBS (pH 7.4) at 37 °C to simulate the conditions used for biological assays on c-CB and c-GB. The c-CI and c-GI hydrogel constructs were printed inside a 24-multiwell plate and characterized on days 1, 7, 14, and 21, through amplitude sweep measurements and diameter assessments. In Figure 4(A), the amplitude sweep curves show that c-CI constructs maintained their gel-like characteristics for 21 days. However, a gradual decline in the moduli was observed on days 7, 14, and 21, indicating a reduction in mechanical properties over time (as summarized in Table 1). Similarly, c-GI constructs preserved their gel-like behavior up to 21 days, as shown in Figure 4(B). Notably, after 1 day, c-GI constructs exhibited higher G′ and G″ moduli, with a crossover occurring at lower shear strain values (from 480% to 47%). This suggests thermal cross-linking, consistent with previous temperature sweep results. Subsequently, construct features remained quite constant over time, with an increase in the Tanδ value observed on day 14 (Table 1), indicating a rise in the viscous component of the material over the solid one. Figure 4(C) illustrates the diameter stability of both constructs: the c-GI construct diameter (red line) showed a 7% reduction in its original size over time, indicating good shape retention under static conditions, while the c-CI construct diameter (blue line) remained relatively constant up to day 14, followed by a sharp 26% decrease at day 21 compared to its initial value probably due to the construct partial degradation. The uniformity ratio values for the constructs on days 1, 7, 14, and 21 were determined by considering the construct diameter and are reported in Figure 4(D): a linear increase in the uniformity ratio of c-GI was observed, associated with the gradual decrease in the diameter over time. The uniformity ratio of c-CI also exhibited a linear increase up to day 14, followed by a sharp rise on day 21. All dimensional measures were performed in triplicate, and SD is reported in the graphs (Figure 4(C,D)).

**Table 1:** Results of Amplitude Sweep Tests: G′ and G″ Values of c-CI and c-GI Constructs after 0, 1, 7, 14, and 21 Days under Static Incubating Conditions at 37 °C

c-CI						c-GI					
	**Day 0**	**Day 1**	**Day 7**	**Day 14**	**Day 21**		**Day 0**	**Day 1**	**Day 7**	**Day 14**	**Day 21**
***G*′ (Pa)**	572	546	306	166	18	***G*′ (Pa)**	96	354	28	45	28
***G*′′ (Pa)**	87.4	59.3	66.1	25.6	5.8	***G*′′ (Pa)**	3.1	10.0	0.8	4.2	2.3
**Tan δ**	0.15	0.11	0.22	0.15	0.31	**Tan δ**	0.03	0.03	0.03	0.09	0.09

### 3.4 Cell Culture and the 3D Bioprinting Process

Before the fabrication of the simplified 3D bioprinted intestinal model, replicating the three fundamental layers of the intestinal barrier, the compatibility ofRPMI 1640 culture medium with both HSFs and HTC-8 cells was assessed. This evaluation aimed to determine the best conditions for coculturing these two cell types, which typically require different culture media. The results are reported in the SI (Figure S2). Each cell type has specific needs according to its function and requires a corresponding specific medium composition. When two or more different cell types are cultured together, choosing the right medium becomes a challenge.^37^ HTCs-8 perfectly grow in RPMI 1640-based medium supplemented with HS, while fibroblasts mainly grow in DMEM-based medium supplemented with FBS. For this reason, several cell culture media mixtures were produced to evaluate the right components of the final formulation, and from the cell proliferation assay, it was evident that HTC-8 can grow only in an RPMI-based medium in the presence of HS. On the contrary, HSFs were found to be able to better support the change of the primary component, from DMEM to RPMI 1640, and the presence of HS in addition to FBS. Indeed, as reported in Figure S2(A), HSFs grew better in the original DMEM-based media even when HS were added to the medium, displaying an increased proliferation rate from 1 RFU at day 1 to 13 RFU at day 21, while when the medium formulation was changed with RPMI 1640 and supplemented with both 5% of HS and 5% of FBS, cell proliferation slightly decreases from 1 RFU at day 1 to 7 RFU at day 21. RPMI 1640 in the copresence of 5% of FBS and 5% of HS was the final optimal formulation to grow both HSFs and HTC-8 cells, which show greater cell proliferation rate with respect to HSFs thanks to the presence of the RPMI (Figure S2(B)). Indeed, the cell proliferation rate of HTC-8 increased from 8 on day 1 to 22 RFU on day 21.

**Figure 5:**
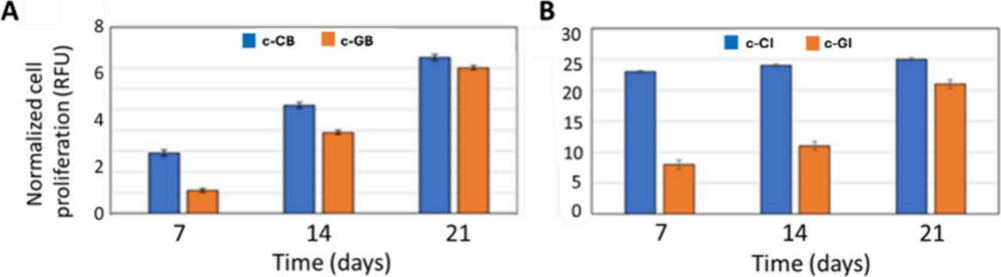
Cell proliferation (A) of HSFs embedded in c-CB and c-GB bioprinted constructs up to 21 days of culture and (B) of HTC-8 growth onto the upper surface of c-CI and c-GI bioprinted constructs up to 21 days of culture.

Then, cell proliferation of both HSFs (after bioprinting and UV cross-linking to form the submucosa layer) and HCT-8 cells (seeded on the 3D construct upper surface to form the epithelial layer) in a 3D hydrogel construct was quantitatively evaluated through alamarBlue assay (Figure 5), in order to assess materials’ compliance with the 3D bioprinting process, in terms of biocompatibility and processing conditions. The proliferation rates of HSFs inside the c-GB and c-CB constructs were found to be similar. Cell growth was higher in the c-CB matrix up to 14 days, but it almost equalized that in c-GB after 21 days. A similar trend was observed for HTC-8 proliferation when seeded onto c-GI and c-CI constructs, with a higher cell growth of collagen matrices. Also, in this case, RFU differences were reduced after 21 days of culture but still in favor of collagen.

**Figure 6:**
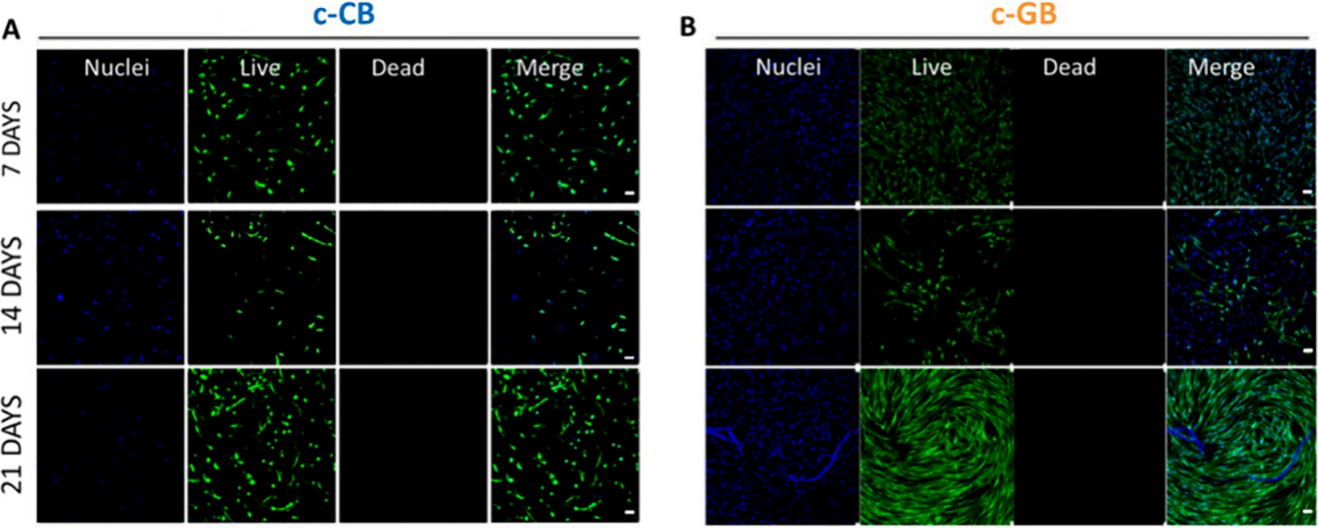
Representative confocal fluorescence images of (A) HSFs embedded in c-CB and (B) c-GB 3D printed constructs after live/dead staining for up to 21 days. Magnification 10×. Scale bar = 50 μm.

The impact of the 3D bioprinting process on HSF survival, as well as the ability of the tested biomaterials (CI and GI) to support cell attachment, spreading, and proliferation over time, were determined *in vitro* through Live/Dead assay up to 21 days. The low degree of cell death after 24 h post printing suggested the compliance of the 3D printing process with cell viability (Figure S3). As regards the ability of proposed materials to support cells processes during time, confocal fluorescence images of HSFs after Live/Dead staining inside the c-CB and c-GB bioprinted constructs at different time points (Figure 6) showed their potential. Homogeneous distribution of live cells (stained with calcein, green color) was observed in both experimental groups (c-CB, Figure 6(A) and c-GB, Figure 6(B)) at day 7 while staining with propidium iodide (dead cells, red color) was minimal, thus indicating that the 3D bioprinting process had a negligible impact on cell survival. Moreover, a clear change in HSF morphology was visible from day 7 to day 21 of incubation, which evolved from round shapes to their typical elongated spindle shapes, especially in c-GB matrices. This can be attributed to the different mechanical properties of scaffolds. c-GI constructs were found to display the lowest stiffness (380 Pa) while c-CI were characterized by a higher value of about 585 Pa. For this reason, HSFs inside c-CB matrices displayed a round-like shape with a minor extent of peripheral filaments (Figure 6(A)). On the contrary, HSFs recognized weaker external forces in c-GB constructs; thus, they retained the ability to form elongations and spiral orientation through the matrix as in 2D condition (Figure 6(B)), where cells appeared flatter with pronounced filaments. Moreover, HSFs in c-CB constructs did not assume spiral orientation, as observed in c-GB. This change in morphology and spatial organization might be due, even in this case, to the different external forces experienced by the cells and provided by the different stiffness of the matrix.

**Figure 7:**
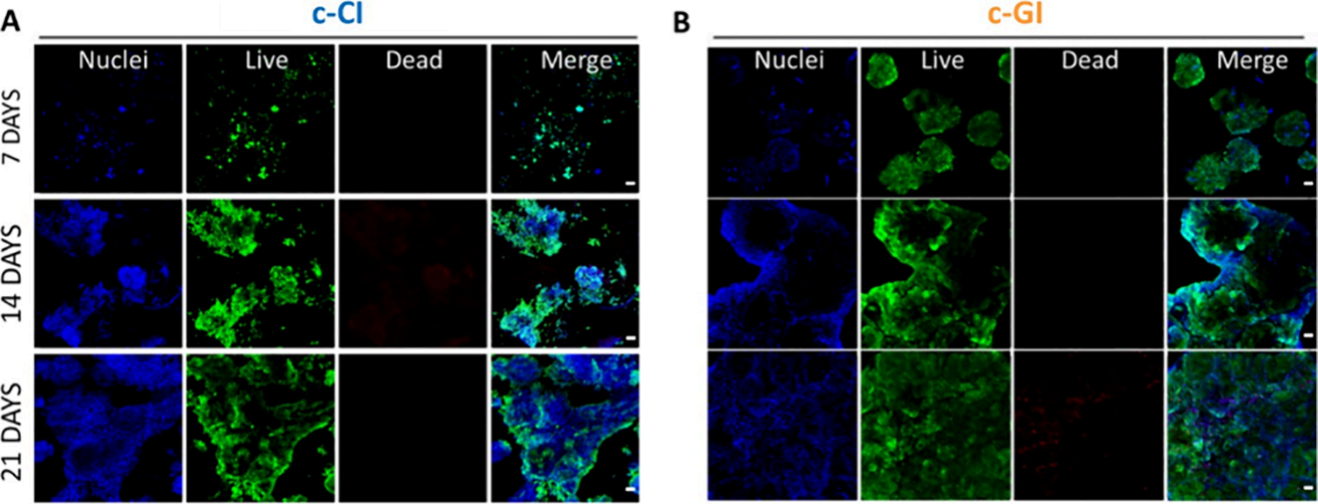
Confocal images of (A) HCT-8 seeded onto c-CI and (B) c-GI 3D bioprinted constructs after live/dead staining up to 21 days. Magnification 10×. Scale bar = 50 μm.

Figure 7 reports confocal fluorescence images after Live/Dead staining of HCTs-8 seeded on c-CI and c-GI 3D bioprinted constructs for up to 21 days of incubation. HTC-8 attached, divided, and formed cell colonies on both materials. However, morphological differences were visible. Cell colonies formed faster on c-GI (after 7 days) rather than on c-CI (after 14 days), because of matrices stiffness. Indeed, HTC-8 tends to form colonies in a stiffness-dependent way. As reported in the literature, this behavior could be ascribed to matrices’ stiffness as well as to the structural conformation of their proteins. It has been documented that HTC-8 tends to form colonies in a stiffness-dependent way.^38,39^ As reported in the work of Tang et al., HTC-8 form cell colonies in 2–4 days on hydrogels with stiffness values of about 21–47 kPa while they retained their round-like shape on matrices with stiffness values of about 0.5–5.0 kPa and occasionally for small colonies.^38^ Indeed, a good level of wetting of HCT-8 was observed on c-GI (0.38 kPa), while poor wetting of HTC-8 was observed on c-CI (0.585 kPa), with a morphological transition from epithelial-like to rounded. However, an augmented wetting was registered during time, with the formation of monolayers and then of colonies in 21 days, suggesting that not only stiffness influences cell response but also protein structural organization since collagen can induce cell adhesion and aggregation.^39^

**Figure 8:**
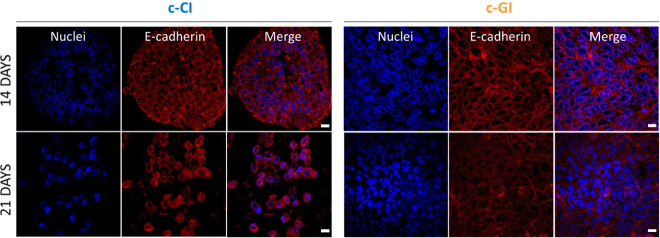
Representative confocal fluorescence images of HCT-8 E-cadherin protein expression after immunostaining with primary and secondary antibodies at days 14 and 21 of culture. Left side shows HCT- 8 protein expression when seeded onto c-CI constructs, while the right side of the image shows HCT-8 protein expression when seeded onto c-GI constructs. Magnification 40×. Scale bar = 10 μm.

**Figure 9:**
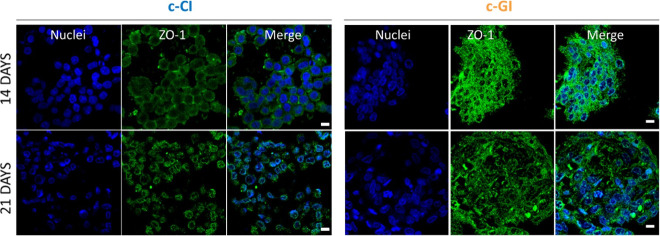
Representative confocal fluorescence images of HCT-8 ZO-1 protein expression after immunostaining with primary and secondary antibodies at days 14 and 21 of culture. Left side shows HCT-8 protein expression when seeded onto c-CB constructs, while the right side of the image show HCT-8 protein expression when seeded onto c-GB constructs. Magnification 40×. Scale bar = 10 μm.

Lastly, the layered structure was fabricated according to Scheme 1 and the integrity of the HCTs-8 monolayer was assessed through the detection of ZO-1 and E-cad protein expression via immunostaining. E-cadherins are a type of cell adhesion molecule that play a key role in the formation of cell-to-cell adherent junctions. Tight junctions are multiprotein junctional complexes whose function is to prevent the leakage of solutes and water and seal the paracellular pathway. Both proteins are typical of the epithelial monolayer, and their presence is fundamental for the replication of a physiologically like intestinal barrier tissue since they strictly influence substance regulation and are responsible for active/passive ion transport.^40^ Confocal fluorescence images reported the E-cadherin expression in red in Figure 8 and the tight junction (ZO-1) in green in Figure 9 of HCTs-8 seeded onto c-GI and c-CI layers at days 14 and 21 of incubation. As is clear from the images, both E-cadherin and tight junctions are produced from the epithelial layer, and they are localized on cells’ intermembrane space. Indeed, it is stated that in physiological conditions, E-cadherins behave as both receptors and ligands for other molecules and, thus are present in the plasma membrane space generated between two or more cells, while tight junctions, which consist of the three major transmembrane proteins such as occludin, claudins, and junction adhesion molecule (JAM) proteins. They are associated with different peripheral membrane proteins such as ZO-1 located on the intracellular side of the plasma membrane, which anchor the strands to the actin component of the cytoskeleton. Furthermore, E-cadherins displayed a strong correlation with cancer since the loss of the cell adhesion molecules is involved in the formation of epithelial types of cancers such as carcinomas. The changes in any type of cadherin expression may not only control tumor cell adhesion but also may affect signal transduction leading to the cancer cells growing uncontrollably. For this reason, the expression of these proteins is fundamental when 3D intestinal barrier models are developed for the in-depth investigation of any type of disease.

## 4 Conclusion

This study evaluates the printability and biological properties of collagen and GelMA hydrogels for developing a simplified intestinal tissue model. The goal of this work is to establish a straightforward protocol for optimizing hydrogel printability in EBB, using affordable and readily available materials. Rheological analysis confirms the shear-thinning behavior of both materials, with u-GI exhibiting higher printability due to its lower flow-index. To replicate all the steps of printing in terms of changes and recovery of the materials’ mechanical properties at different applied shear rates, the 3ITT test was conducted. It is noteworthy that the printability indexes derived from the fitting of flow curves are consistent with the results obtained from the 3ITT analysis and with the parameters calculated through the dimensional evaluation of printed filaments and constructs. u-GI demonstrates higher printability compared to u-CI, as evidenced by the lower flow index value of the former, indicating a more pronounced shear-thinning characteristic, although both materials exhibit good flow index values. This finding is confirmed by the printability factor, which is closer to 1 for c-GI compared to c-CI. While u-CI shows better recovery of mechanical properties postprinting (85% vs 45% for u-GI), c-GI constructs better maintain structural integrity during incubation. Additionally, c-GI’s higher filament roughness could be beneficial for mimicking intestinal tissue. Finally, cell viability, cell proliferation, and immunostaining assays allowed the assessment of the biological properties of the employed materials and to achieve a first validation of the developed intestinal model. Both hydrogels supported cell proliferation and viability, with slight differences observed in cell growth rates over time. Collagen matrices resulted in higher cell proliferation initially, but differences diminished over 21 days. Results demonstrated that both the biomaterials and the 3D bioprinting method employed herein enabled the fabrication of trilayer intestinal models composed of fibroblasts and intestinal epithelial cells in the submucosa and the epithelial layers, respectively, ensuring high cell survival and promoting the proliferation and the expression of proteins supporting the formation of the intestinal barrier. This model bridges the gap between simplistic 2D cultures and clinical applications, aligning with the 3Rs principle to reduce animal testing. Beyond the proof-of-concept, this platform holds promise for studying barrier function, permeability, metabolism, and drug testing. Future improvements could enhance the mechanical stability, optimize cell–material interactions, and integrate immune cells or microbiota to enhance physiological relevance. Overall, collagen- and GelMA-based bioprinted hydrogels offer a promising approach for intestinal tissue engineering and therapeutic research.

## Supporting Information Available

Printed flower pattern of c-CI and c-GI compared to the CAD model; 2D cell proliferation of HSFs in DMEM and RPMI-based cell culture medium after 1, 7, 14, and 21 days of incubation; 2D cell proliferation of HCTs-8 in RPMI-based medium after 1, 7, 14, and 21 days of incubation; representative confocal fluorescence images of HSFs embedded in c-CB and c-GB 3D printed constructs after live/dead staining after 24 h (PDF)

## References

[ref1] Tan B.; Gan S.; Wang X.; Liu W.; Li X. Applications of 3D bioprinting in tissue engineering: advantages, deficiencies, improvements, and future perspectives. J. Mater. Chem. B 2021, 9, 5385–5413. 10.1039/D1TB00172H. 34124724

[ref2] Pagnotta G.; Kalia S.; Di Lisa L.; Cicero A. F. G.; Borghi C.; Focarete M. L. Progress towards 3D bioprinting of tissue models for advanced drug screening: In vitro evaluation of drug toxicity and drug metabolism. Bioprinting 2022, 27, e00218 10.1016/j.bprint.2022.e00218.

[ref3] Bhise N. S.; Manoharan V.; Massa S.; Tamayol A.; Ghaderi M.; Miscuglio M.; Lang Q.; Shrike Zhang Y.; Shin S. R.; Calzone G.; Annabi N.; Shupe T. D.; Bishop C. E.; Atala A.; Dokmeci M. R.; Khademhosseini A. A liver-on-a-chip platform with bioprinted hepatic spheroids. Biofabrication 2016, 8, 014101 10.1088/1758-5090/8/1/014101. 26756674

[ref4] Pati F.; Gantelius J.; Svahn H. A. 3D Bioprinting of Tissue/Organ Models. Angew. Chem., Int. Ed. 2016, 55, 4650–65. 10.1002/anie.201505062. 26895542

[ref5] Ozbolat I. T.; Peng W.; Ozbolat V. Application areas of 3D bioprinting. Drug Discov Today 2016, 21, 1257–71. 10.1016/j.drudis.2016.04.006. 27086009

[ref6] Peng W.; Unutmaz D.; Ozbolat I. T. Bioprinting towards Physiologically Relevant Tissue Models for Pharmaceutics. Trends Biotechnol 2016, 34, 722–32. 10.1016/j.tibtech.2016.05.013. 27296078

[ref7] Vanaei S.; Parizi M. S.; Vanaei S.; Salemizadehparizi F.; Vanaei H. R. An Overview on Materials and Techniques in 3D Bioprinting Toward Biomedical Application. Engineered Regeneration 2021, 2, 1–18. 10.1016/j.engreg.2020.12.001.

[ref8] Vijayavenkataraman S.; Yan W.-C.; Lu W. F.; Wang C.-H.; Fuh J. Y. H. 3D bioprinting of tissues and organs for regenerative medicine. Adv. Drug Deliv Rev. 2018, 132, 296–332. 10.1016/j.addr.2018.07.004. 29990578

[ref9] Torras N.; Zabalo J.; Abril E.; Carré A.; García-Díaz M.; Martínez E. A bioprinted 3D gut model with crypt-villus structures to mimic the intestinal epithelial-stromal microenvironment. Biomaterials Advances 2023, 153, 213534 10.1016/j.bioadv.2023.213534. 37356284

[ref10] Tian S.; Zhao H.; Lewinski N. Key parameters and applications of extrusion-based bioprinting. Bioprinting 2021, 23, e00156 10.1016/j.bprint.2021.e00156.

[ref11] Schwab A.; Levato R.; D’Este M.; Piluso S.; Eglin D.; Malda J. Printability and Shape Fidelity of Bioinks in 3D Bioprinting. Chem. Rev. 2020, 120, 11028–55. 10.1021/acs.chemrev.0c00084. 32856892 PMC7564085

[ref12] Rezvani Ghomi E.; Nourbakhsh N.; Akbari Kenari M.; Zare M.; Ramakrishna S. Collagen-based biomaterials for biomedical applications. J. Biomed Mater. Res. B Appl. Biomater 2021, 109, 1986–99. 10.1002/jbm.b.34881. 34028179

[ref13] Sorushanova A.; Delgado L. M.; Wu Z.; Shologu N.; Kshirsagar A.; Raghunath R.; Mullen A. M.; Bayon Y.; Pandit A.; Raghunath M.; Zeugolis D. I. The Collagen Suprafamily: From Biosynthesis to Advanced Biomaterial Development. Adv. Mater. 2019, 31 (1), e1801651 10.1002/adma.201801651. 30126066

[ref14] Lee J. M.; Suen S. K. Q.; Ng W. L.; Ma W. C.; Yeong W. Y. Bioprinting of Collagen: Considerations, Potentials, and Applications. Macromol. Biosci. 2021, 21, 10.1002/mabi.202000280. 33073537

[ref15] O’Connell C. D.; Zhang B.; Onofrillo C.; Duchi S.; Blanchard R.; Quigley A.; Bourke J.; Gambhir S.; Kapsa R.; Di Bella C.; Choong P.; Wallace G. G. Tailoring the mechanical properties of gelatin methacryloyl hydrogels through manipulation of the photocrosslinking conditions. Soft Matter 2018, 14, 2142–51. 10.1039/C7SM02187A. 29488996

[ref16] Ying G.; Jiang N.; Yu C.; Zhang Y. S. Three-dimensional bioprinting of gelatin methacryloyl (GelMA). Biodes Manuf 2018, 1, 215–24. 10.1007/s42242-018-0028-8.

[ref17] Vancamelbeke M.; Vermeire S. The intestinal barrier: a fundamental role in health and disease. Expert Rev. Gastroenterol Hepatol 2017, 11, 821–34. 10.1080/17474124.2017.1343143. 28650209 PMC6104804

[ref18] Prashantha K.; Krishnappa A.; Muthappa M. 3D bioprinting of gastrointestinal cancer models: A comprehensive review on processing, properties, and therapeutic implications. Biointerphases 2023, 18, 10.1116/6.0002372. 36963961

[ref19] Macedo M. H.; Martínez E.; Barrias C. C.; Sarmento B. Development of an Improved 3D in vitro Intestinal Model to Perform Permeability Studies of Paracellular Compounds. Front. Bioeng. Biotechnol. 2020, 8, 10.3389/fbioe.2020.524018. PMC7527803 33042961

[ref20] Elomaa L.; Gerbeth L.; Almalla A.; Fribiczer N.; Daneshgar A.; Tang P.; Hillebrandt K.; Seiffert S.; Sauer I. M.; Siegmund B.; Weinhart M. Bioactive photocrosslinkable resin solely based on refined decellularized small intestine submucosa for vat photopolymerization of in vitro tissue mimics. Addit Manuf 2023, 64, 103439 10.1016/j.addma.2023.103439.

[ref21] Sbirkov Y.; Molander D.; Milet C.; Bodurov I.; Atanasov B.; Penkov R.; Belev N.; Forraz N.; McGuckin C.; Sarafian V. A Colorectal Cancer 3D Bioprinting Workflow as a Platform for Disease Modeling and Chemotherapeutic Screening. Front. Bioeng. Biotechnol. 2021, 9. 10.3389/fbioe.2021.755563. PMC8638705 34869264

[ref22] Castro F.; Leite Pereira C.; Helena Macedo M.; Almeida A.; JoséSilveira M.; Dias S.; Patrícia Cardoso A.; JoséOliveira M.; Sarmento B. Advances on colorectal cancer 3D models: The needed translational technology for nanomedicine screening. Adv. Drug Deliv Rev. 2021, 175, 113824 10.1016/j.addr.2021.06.001. 34090966

[ref23] Creff J.; Courson R.; Mangeat T.; Foncy J.; Souleille S.; Thibault C.; Besson A.; Malaquin L. Fabrication of 3D scaffolds reproducing intestinal epithelium topography by high-resolution 3D stereolithography. Biomaterials 2019, 221, 119404 10.1016/j.biomaterials.2019.119404. 31419651

[ref24] Mazzaglia C.; Sheng Y.; Rodrigues L. N.; Lei I. M.; Shields J. D.; Huang Y. Y. S. Deployable extrusion bioprinting of compartmental tumoroids with cancer associated fibroblasts for immune cell interactions. Biofabrication 2023, 15, 025005 10.1088/1758-5090/acb1db. 36626838

[ref25] Han H.; Park Y.; Choi Y.; Yong U.; Kang B.; Shin W.; Min S.; Kim H. J.; Jang J. A Bioprinted Tubular Intestine Model Using a Colon-Specific Extracellular Matrix Bioink. Adv. Healthcare Mater. 2022, 11, 10.1002/adhm.202101768. 34747158

[ref26] Kim W.; Kim G. H. An intestinal model with a finger-like villus structure fabricated using a bioprinting process and collagen/SIS-based cell-laden bioink. Theranostics 2020, 10, 2495–508. 10.7150/thno.41225. 32194815 PMC7052892

[ref27] Taebnia N.; Zhang R.; Kromann E. B.; Dolatshahi-Pirouz A.; Andresen T. L.; Larsen N. B. Dual-Material 3D-Printed Intestinal Model Devices with Integrated Villi-like Scaffolds. ACS Appl. Mater. Interfaces 2021, 13, 58434–46. 10.1021/acsami.1c22185. 34866391

[ref28] Almutary A. G.; Alnuqaydan A. M.; Almatroodi S. A.; Bakshi H. A.; Chellappan D. K.; Tambuwala M. M. Development of 3D-Bioprinted Colitis-Mimicking Model to Assess Epithelial Barrier Function Using Albumin Nano-Encapsulated Anti-Inflammatory Drugs. Biomimetics 2023, 8, 41 10.3390/biomimetics8010041. 36810372 PMC9944493

[ref29] Clevers H. The Intestinal Crypt, A Prototype Stem Cell Compartment. Cell 2013, 154, 274–84. 10.1016/j.cell.2013.07.004. 23870119

[ref30] Shirahama H.; Lee B. H.; Tan L. P.; Cho N.-J. Precise Tuning of Facile One-Pot Gelatin Methacryloyl (GelMA) Synthesis. Sci. Rep 2016, 6, 31036 10.1038/srep31036. 27503340 PMC4977492

[ref31] López de Andrés J.; Ruiz-Toranzo M.; Antich C.; Chocarro-Wrona C.; López-Ruíz E.; Jiménez G.; Marchal J. A. Biofabrication of a tri-layered 3D-bioprinted CSC-based malignant melanoma model for personalized cancer treatment. Biofabrication 2023, 15, 035016 10.1088/1758-5090/ac8dc6. 36041423

[ref32] Dominici M.; Le Blanc K.; Mueller I.; Slaper-Cortenbach I.; Marini F. C.; Krause D. S.; Deans R. J.; Keating A.; Prockop D. J.; Horwitz E. M. Minimal criteria for defining multipotent mesenchymal stromal cells. The International Society for Cellular Therapy position statement. Cytotherapy 2006, 8, 315–7. 10.1080/14653240600855905. 16923606

[ref33] Garreta E.; de Oñate L.; Fernández-Santos M. E.; Oria R.; Tarantino C.; Climent A. M.; Marco A.; Samitier M.; Martínez E.; Valls-Margarit M.; Matesanz R.; Taylor D. A.; Fernández-Avilés F.; Izpisua Belmonte J. C.; Montserrat N. Myocardial commitment from human pluripotent stem cells: Rapid production of human heart grafts. Biomaterials 2016, 98, 64–78. 10.1016/j.biomaterials.2016.04.003. 27179434

[ref34] Lemarié L.; Anandan A.; Petiot E.; Marquette C.; Courtial E.-J. Rheology, simulation and data analysis toward bioprinting cell viability awareness. Bioprinting 2021, 21, e00119 10.1016/j.bprint.2020.e00119.

[ref35] Blaeser A.; Duarte Campos D. F.; Puster U.; Richtering W.; Stevens M. M.; Fischer H. Controlling Shear Stress in 3D Bioprinting is a Key Factor to Balance Printing Resolution and Stem Cell Integrity. Adv. Healthc Mater. 2016, 5, 326–33. 10.1002/adhm.201500677. 26626828

[ref36] Zhao C.; Wu Z.; Chu H.; Wang T.; Qiu S.; Zhou J.; Zhu Q.; Liu X.; Quan D.; Bai Y. Thiol-Rich Multifunctional Macromolecular Crosslinker for Gelatin-Norbornene-Based Bioprinting. Biomacromolecules 2021, 22, 2729–39. 10.1021/acs.biomac.1c00421. 34057830

[ref37] Vis M. A. M.; Ito K.; Hofmann S. Impact of Culture Medium on Cellular Interactions in in vitro Co-culture Systems. Front. Bioeng. Biotechnol. 2020, 8, 10.3389/fbioe.2020.00911. PMC7417654 32850750

[ref38] Tang X.; Kuhlenschmidt T. B.; Zhou J.; Bell P.; Wang F.; Kuhlenschmidt M. S.; Saif T. A. Mechanical Force Affects Expression of an In Vitro Metastasis-Like Phenotype in HCT-8 Cells. Biophys. J. 2010, 99, 2460–9. 10.1016/j.bpj.2010.08.034. 20959086 PMC2955412

[ref39] Ali M. Y.; Chuang C.-Y.; Saif M. T. A. 2014 Reprogramming cellular phenotype by soft collagen gels. Soft Matter 2014, 10, 8829–37. 10.1039/C4SM01602E. 25284029 PMC4208984

[ref40] Itoh M.; Nagafuchi A.; Moroi S.; Tsukita S. Involvement of ZO-1 in Cadherin-based Cell Adhesion through Its Direct Binding to α Catenin and Actin Filaments. J. Cell Biol. 1997, 138, 181–92. 10.1083/jcb.138.1.181. 9214391 PMC2139940

