# Supplementary material for: Establishing a Bioink Assessment Protocol: GelMA and Collagen in the Bioprinting of a Potential In Vitro Intestinal Model
Source: ACS Biomater Sci Eng. 2025 Mar 25;11(4):2456–67. doi: 10.1021/acsbiomaterials.5c00034 (PMC12001187; doi:10.1021/acsbiomaterials.5c00034)
Supplement: Supplementary file 1 — ab5c00034_si_001.pdf [file ab5c00034_si_001.pdf]

Supporting Information

Establishing a bioink assessment protocol: GelMA and Collagen in the bioprinting of a potential *in vitro* intestinal model

Mariangela Rea[1,#], Luana Di Lisa[1,#], Giorgia Pagnotta[1], Nunzia Gallo[2,3], Luca Salvatore[3], Federica D'Amico[4], Noelia Campilio[5], José Manuel Baena[5,6], Juan Antonio Marchal[7,8,9,10,*], Arrigo F.G. Cicero[11,12], Claudio Borghi[11,12], Maria Letizia Focarete[1,13,*]

[1] Department of Chemistry 'Giacomo Ciamician' and INSTM UdR of Bologna, University of Bologna, 40129 Bologna, Italy
[2] Department of Engineering for Innovation, University of Salento, 73100 Lecce, Italy
[3] Typeone Biomaterials S.r.l., Via Europa 167, 73021 Calimera, Lecce, Italy
[4] Department of Pharmacy and Biotechnology, University of Bologna, 40126 Bologna, Italy
[5] REGEMAT 3D S.L. 18016, Granada Spain
[6] BRECA Health Care S.L., 18016, Granada, Spain
[7] Department of Human Anatomy and Embryology, Faculty of Medicine, University of Granada, 18016 Granada, Spain.
[8] BioFab i3D Lab, Centre for Biomedical Research (CIBM), University of Granada, 18016 Granada, Spain
[9] Instituto de Investigación Biosanitaria ibs.GRANADA, 18016 Granada, Spain.
[10] Excellence Research Unit "Modeling Nature" (MNat), University of Granada, 18071 Granada, Spain.
[11] Medical and Surgery Sciences Dept., University of Bologna, 40138 Bologna, Italy
[12] Cardiovascular Medicine Unit, IRCCS AOU di Bologna, 40138 Bologna, Italy
[13] Interdepartmental Center for Industrial Research in Health Sciences and Technologies, University of Bologna, Via Tolara di Sopra, 41/E, 40064 Ozzano Emilia, Bologna, Italy

[#] The authors equally contributed to this work

* E-mail:
jmarchal@go.ugr.es
marialetizia.focarete@unibo.it

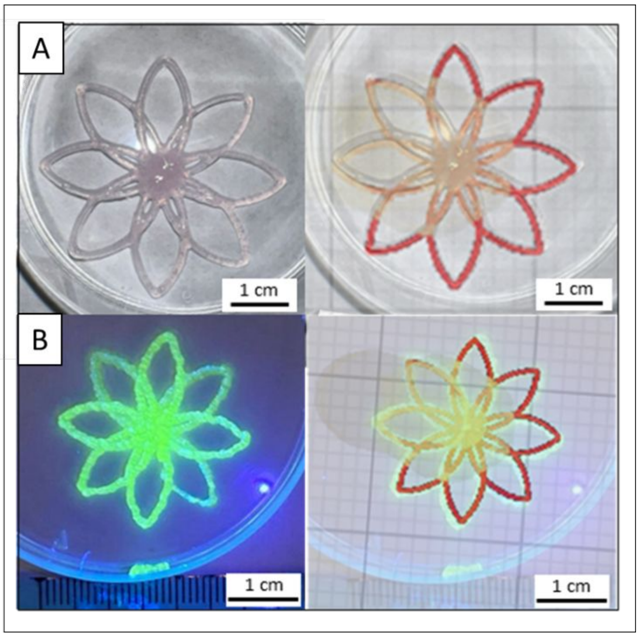

Figure S1. (A) Printed flower pattern of c-CI (on the left) and superposition with the CAD model (on the right); (B) Printed flower pattern of c-GI (on the left) compared to the CAD model (on the right).

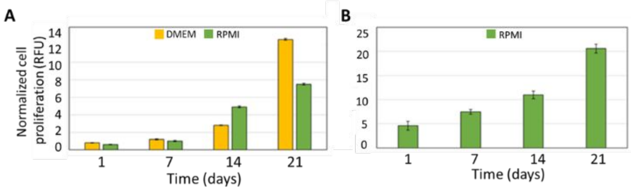

Figure S2. (A) 2D cell proliferation of HSFs in DMEM (yellow bars) and in RPMI-based (green bars) cell culture medium after 1, 7, 14, and 21 days of incubation (B) 2D cell proliferation of HCTs-8 in RPMI-based medium after 1, 7, 14, and 21 days of incubation. Results are reported as mean +/- SD, n=3.

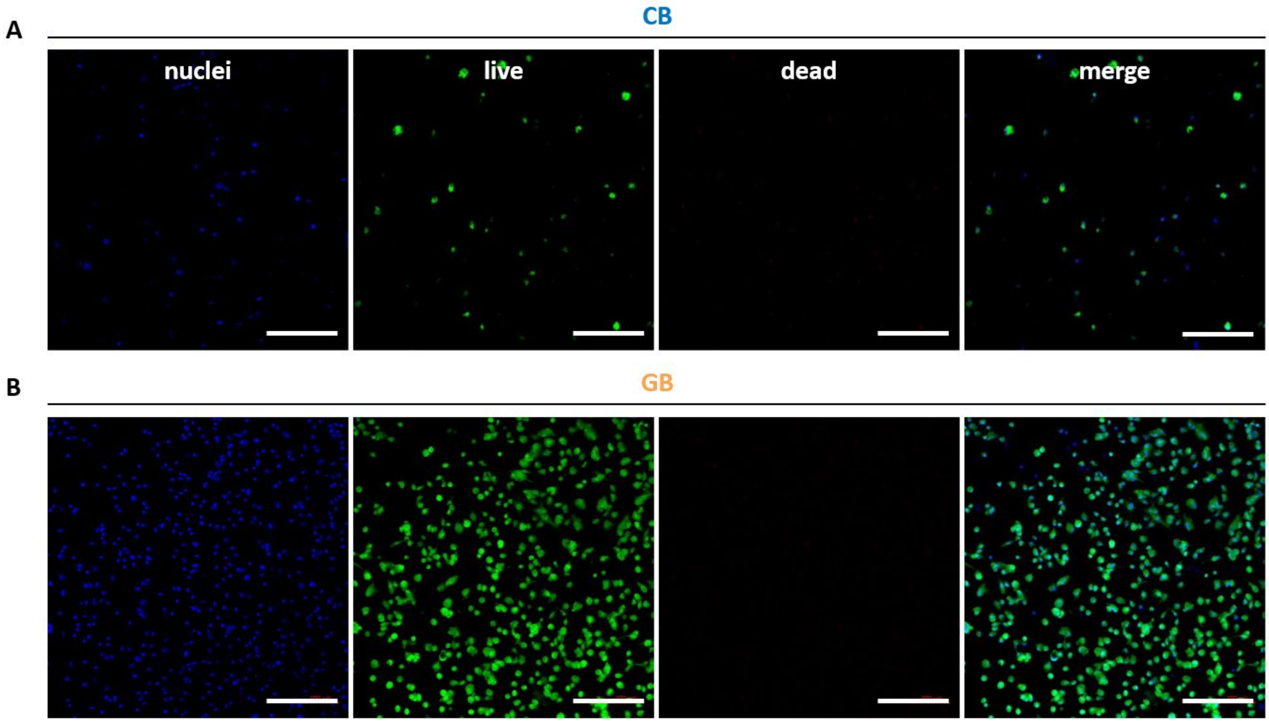

Figure S3. Representative confocal fluorescence images of HSFs embedded in c-CB (A) and in c-GB (B) 3D printed constructs after live/dead staining after 24h. Magnification 10X. Scale bar = 200 µm.
